# Supplementary material for: Predicting visual function by interpreting a neuronal wiring diagram
Source: Nature. 2024 Oct 2;634(8032):113–23. doi: 10.1038/s41586-024-07953-5 (PMC11446822; doi:10.1038/s41586-024-07953-5)
Supplement: Supplementary file 6 — CRF and ERF predictions for individual TmY4 and TmY9 cells. Analogous to Supplementary Data 3, but for TmY target types. Shown are the top four monosynaptic pathways, the strongest pathway passing through each of the top ten intermediary types (ranking from Extended Data Fig. 7), and the trisynaptic pathway Tm1–TmY–Dm3–TmY (see the section entitled Prediction of spatial normalization). [file 41586_2024_7953_MOESM6_ESM.zip › DataS4/TmY9qΓèÑ/720575940626020666.pdf]

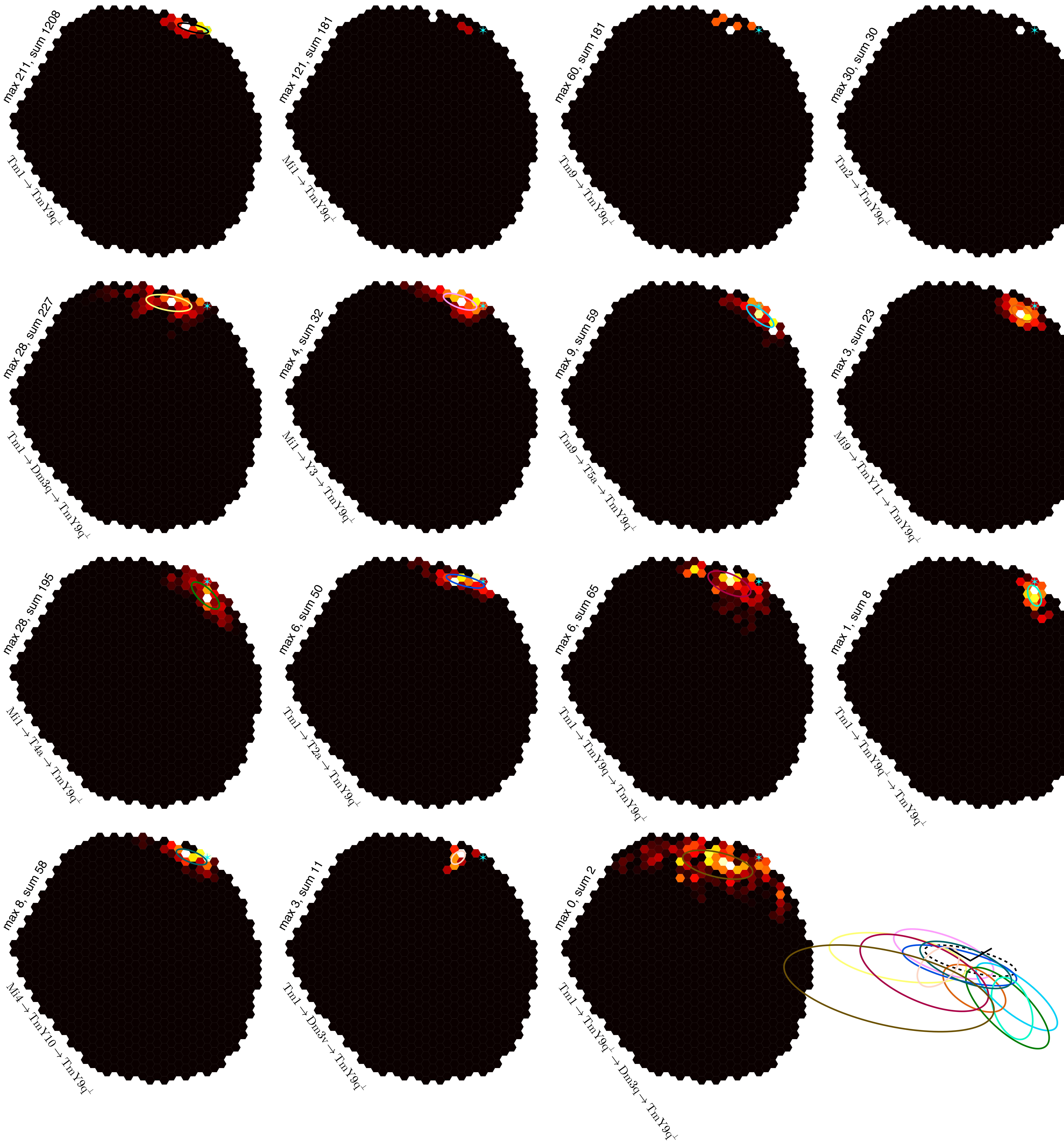
max 211, sum 1208
Tm1 → TmY9q⊥
max 121, sum 181
Mi1 → TmY9q⊥
max 60, sum 181
Tm9 → TmY9q⊥
max 30, sum 30
Tm2 → TmY9q⊥
max 28, sum 227
Tm1 → Dm3q → TmY9q⊥
max 4, sum 32
Mi1 → Y3 → TmY9q⊥
max 9, sum 59
Tm9 → T5a → TmY9q⊥
max 3, sum 23
Mi9 → TmY11 → TmY9q⊥
max 28, sum 195
Mi1 → T4a → TmY9q⊥
max 6, sum 50
Tm1 → T2a → TmY9q⊥
max 6, sum 65
Tm1 → TmY9q → TmY9q⊥
max 1, sum 8
Tm1 → TmY9q⊥ → TmY9q⊥
max 8, sum 58
Mi4 → TmY10 → TmY9q⊥
max 3, sum 11
Tm1 → Dm3v → TmY9q⊥
max 0, sum 2
Tm1 → TmY9q⊥ → Dm3q → TmY9q⊥
